# The Study of the Urine without a Microscope1A clinical lecture to the nurses of the Buffalo Hospital Sisters of Charity, May, 1902.

**Published:** 1902-10

**Authors:** Byron H. Daggett

**Affiliations:** Genitourinary Surgeon Buffalo Hospital Sisters of Charity, Erie County, St. Mary’s Infant and Maternity, and Riverside Hospitals. 723 Ellicott Square.


### CLINICAL LECTURE.

### The Study of the Urine Without a Microscope.[1]

By BYRON H. DAGGETT, M. D.

Genitourinary Surgeon Buffalo Hospital Sisters of Charity, Erie County, St. Mary's Infant and Maternity, and Riverside Hospitals.

MACROSCOPICAL examination of urine furnishes valuable data in a clinical record. Such an examination is easily done and reveals clinical questions otherwise unattainable. No great amount of technical skill is required, no complicated apparatus is necessary. The results of such an examination are plain and easily understood. The urine of the male should be collected in an open-mouthed bottle, and that of the female, when accurate analysis is necessary, should be drawn by the catheter.

If urine is to be examined within twenty-four hours of its passage, antiseptics are not required, but if kept longer than a day some antiseptic, as thymol or chloral, should be added—only a few grains are necessary.

In all cases where the urine escapes in drops, or none is passed, the question of retention or suppression should be decided by passing a catheter or by percussion. These means differentiate between retention and suppression.

*Quantity.*—The amount of urine passed in one day should be determined by measurement, and repeated at intervals, in all important cases. The urine should be examined as a routine measure in all cases. Kidney diseases existing for unknown periods, unsuspected by the physician in attendance, have been subsequently revealed by careful examination of the urine.

A healthy adult of average weight will pass from 40 to 60 ounces per day—1200 to 1800 c.c.

Cold, digestion of large quantities of fluids will largely increase the daily amount. Catharsis, skin temperature, vascular conditions, vaso-motor disturbances, nephritic diseases, choked condition of the kidneys from metabolic products, irritants and poisons decrease the amount passed.

*Color.*—Normal urine is the color of amber or light straw. Neubauer and Vogel give a scale of nine colors ranging from a pale yellow to a brownish black, which are due to natural pigment or admixture of foreign coloring matter. The peculiar color of normal urine is due to a nitrogenised principle known

1. A clinical lecture to the nurses of the Buffalo Hospital Sisters of Charity, May, 1902.

by a variety of names, viz.: urrosaccin, urochrom, urohemetin, uroxanthin and purpurin.

Its ultimate composition is not settled, although it contains C.O.H., N and probably iron. It is supposed to be much like the coloring matter of the blood, and probably formed from hematin, the coloring matter of that fluid.

Blood tinges urine from a smoky hue to many hues of red; bile and santomin make it yellow; rhubard, brown; carbolic acid, smoky, and methylin blue an intense blue color; sulphur browns the urine; rheumatism and fevers make it brown.

*Odor.*—The odor of fresh urine is slightly aromatic; its cause is unknown. The temperature is nearly 100° F. when passing. The use of certain foods and medicines change its odor remarkably. For example, asparagus, cauliflower, turpentine and the balsams. Bile pigment gives it a faint odor of chloroform, and it has a putrid odor when intense intestinal putrefaction is going on. Its decomposition begets an odor of ammonia and alkaline reaction, if it occurs in the bladder; acid reaction if the pathologic condition occurs in the kidneys.

*Transparency.*—Normal urine is always clear—transparent. To determine whether it is transparent or not, you must use a test-tube or a glass vessel. You cannot decide this quality by looking into or upon the fluid in an opaque vessel. You must look through it.

*Reaction.*—Normal urine is acid in reaction, and its acidity is due especially to the acid alkali-phosphate. It may be due to the presence of a free organic acid, but the rôle played by free acid is unimportant, and the fact of its presence is a mooted question. Sometimes directly after a meal the urine is alkaline, but this phenomenon soon disappears, and it has no clinical significance.

The alkaline carbonates and salts of the vegetable acids taken into the stomach render the urine alkaline. It may seem curious that both alkalies and vegetable acids have this effect, but physiological chemistry accounts for this phenomenon.

The reaction of urine is usually tested by blue and red litmus paper. You must note whether the urine is alkaline when voided, and after standing for a time. Note also whether the alkalinity, if present, is due to the volatile (ammo. carb. from breaking up of urea), or the fixed alkalies (alkali-phosphates) taken into the body as food or medicine. To determine this point, the red litmus, turned blue by the urine if exposed for a time to warm air, will return to its red color, but if the reaction is from fixed alkalies it will remain blue after drying. Sometimes the same urine will turn blue litmus red, and red litmus blue. This phenomenon is called amphoteric, and has no satisfactory explanation and no symptomatic value, so far as we know.

Bradshaw, of Liverpool, maintains that litmus should not be employed for testing the reaction of the urine, because solutions of the salts of phosphoric acid are never neutral to litmus; that if the urine is not distinctly acid or alkaline it is amphoteric.

For this reason Bradshaw advocated the use of phenolphthalein as an indicator of acidity, and maintained that objections to its use are not valid. The teaching is that acidity of the urine is diminished after meals. Bradshaw found, after making 160 estimates of the acidity of urine, that in health the acidity followed a fairly regular curve; high during sleep, it fell rapidly after "getting up," and remained at a minimum until about noon, after which it rose steadily and reached a maximum about 9 p.m.

He claims that the degree of acidity is apparently diminished or increased by a more or less bulk of water present, and that alkalies and other diuretics only apparently lessen the amount of acid by dilution and by combination.

*Specific Gravity.*—In normal urine there are about 967 parts of water and 33 of solids to the 1000, specific gravity about 1,020; this quality varies from 1015 to 1030 within the range of health. As a rule, the lighter the color the greater the quantity passed within 24 hours. A high gravity indicates a large amount of solids—a low specific gravity indicates a diminished amount of solids. A large amount of solids indicates great activity of the granular epithelial elements of the kidney. The amount of solids excreted in 24 hours is from 60 to 70 grams—960 to 1,120 grains.

Urinary insufficiency is a condition in which the amount of solids is abnormally diminished, without reference to the bulk of water. The kidneys are the great safety valves of the system, filtering from the blood the poisonous waste of the living, active organs of the body, as well as any foreign substance found in the blood.

The waste, or excrementitious matter of the body is held in solution in the urine and consists of nitrogenous elements and salts. We call them solids to distinguish between them and their menstruum.

The solids are the distinguishing characteristics of urine. They make urine heavier than water, hence the importance of knowing the specific gravity of urine, and as it varies from hour to hour to know the gravity of a 24-hour product.

A ready rule for determining the total excretion of solids is as follows: multiply the last two figures of the gravity by the number of ounces passed in 24 hours, and this product by 1.1; this will approximately give the total solids in grains, and a good estimate of the excretory activity of the kidneys.

Solids of human urine, according to Flint, are: (1) urea; (2) urate of soda, neutral and acid; urate of ammonia, neutral and acid, small quantity: urate of potassa, a trace; urate of lime, a trace; urate of magnesia a trace; hipporate of soda, potassa and lime; lactate of soda, potassa and lime; creatin; creolin-oxalate of lime; xanthin; chloride of sodium; chloride of potassium, hydro-chlorate of ammonia; sulphate of soda, potassa and lime, acid and basic; phosphate of magnesia; ammonia-magnesium phosphate; silicic acid; urrosaccin, coloring matter; mucus and gases.

The urea and chlorides are present in a large percentage, so that the specific gravity is mostly due to these substances. The other substances are present in small quantities and some only in traces.

Urea is the most constant of the urinary constituents, and exists in the greatest quantity. It is one of the most interesting constituents of the urine—it rids the body of 90 per cent of its waste nitrogen. "It is the most important end product of proteid metabolism." 20 to 40 grams are excreted each day. Urea forms 2 per cent of the normal urine. It is probably formed in the liver, at least it is much decreased in quantity in atrophy of the liver.

It is increased by cold bathing, exercise, and a meat diet. It diminishes in disease attended by emaciation. It disappears from the urine in uremia. Its diminution indicates epithelial impermeability of the glandular epithelium of the kidney, and if persistent it indicates Bright's disease.

The retention of urea arrests the osmotic processes of the cells, elimination ceases and so-called uremia results from an accumulation of the end products of proteid metabolism. Urea is the natural diuretic, yet if it fails to perform its function of elimination of by-products, it is indirectly poisonous. Urea is found in several fluids and solids—muscles and liver—of the body.

It is a nitrogenous substance and is derived from the waste of nitrogenous matter. It is one of the few organic principles that can be produced synthetically by the chemist.

Uric acid, creatin, creatinin are other products of proteid metabolism. Uric acid is probably produced by the liver; it is non-poisonous and has no clinical significance. It is more important as an indicator of other processes than its own formation and presence would signify.

Uric acid has been given as a cause of many maladies, but recent investigations indicate that it is not so bad after all. It does not cause gout, rheumatism or neuralgia. It is not a strong poison, for sufficient alkali to neutralise its effect is more harmful to protoplasm than the acid itself. It may deposit spontaneously from urine allowed to stand a few hours or upon the addition of hydrochloric acid. There is no simple clinical process for its elimination. Its proportion to urea is 1 to 30, 1 to 100, and Haig says 1 to 350 in extreme cases.

Indican is an ingredient of normal urine. It is the product of intestinal putrefaction. It is increased in all conditions which beget intestinal decomposition—appendicitis, peritonitis, intussusception, and the like. There are other aromatic products of intestinal disturbance whose importance cannot be determined.

The ethereal sulphates are derived from proteid putrefaction; the other sulphates maintain their normal relation with urea. There are from 10 to 15 grains of chloride of sodium excreted each day. This amount is diminished in pneumonia and fevers.

The chlorides are next in amount to urea. They are almost entirely the potassic and sodic salts. They diminish in repose of the body, in all acute febrile diseases and in chronic affections. They increase with a salty diet, with energetic bodily or mental exercise, in intermittent fever and dropsy. The normal inorganic constituents are taken into the system with the food.

Three and one-half grams of phosphoric acid in combination are excreted daily. The phosphates are increased in febrile conditions by animal food, by the introduction of phosphatic compounds. They are decreased in urine of low gravity in kidney and heart diseases, in disorders of digestion and chronic brain diseases.

Blood may find its way into the urine from the kidneys, bladder, or urethra. Blood pigments, from the destruction of the blood cells in the circulation, may appear in the urine. Both darken the urine. The presence of blood in the urine may be shown by putting into it a few drops of the fresh tincture guaiac, which gives it a faint blue color.

In addition to the normal constituents, there are often found in urine abnormal accidental substances which change its appearance and character.

The urine is a complex fluid; as I have intimated, there is yet much to be learned about its character and composition. It contains poisonous and other qualities not yet revealed to human knowledge. Fresh, normal urine is not baneful, but it readily changes or decomposes, creating a insidious poison.

The urine is one of the most reliable guides for estimating the variations of waste and nutrition. All diseases can not be diagnosticated by urinary examination, yet no serious pathological condition can exist for any length of time without creating some urinary change, some manifestation of disease.

By frequent examination of the urine the physician may know whether his case is gaining or losing ground. Life insurance companies exact a careful chemical and microscopical examination of the urine before issuing policies for insurance. Surgeons require examination of the urine before attempting important operations. During the latter months of pregnancy the urine should be examined once each month and the last month once a week, in order to be forwarned of danger. Deficiency of urea signalises the approach of an eclamptic storm.

These clinical data are therapeutic guide posts along the medical highway: (1) Color, (2) odor, (3) reaction—neutral, acid, alkaline, (4) specific gravity—1010 to 1028, (5) quantity—24 hours' product, (6) amount of solids—8 to 1200 grs., (7) albumin, (8) blood, (9) pus, (10) sugar.

723 Ellicott Square.

---

The older methods of treatment for epistaxis (*Clinical Review*) while ordinarily reasonably positive and satisfactory, must give way to the newer method of the employment of the extract of the suprarenal gland. Not only is the application of this solution followed by almost instantaneous blanching of the mucous membrane and stoppage of the hemorrhage, but it has a very wide range of effect. Except in aged persons with a cardiopathic condition causing the epistaxis the method is universally applicable and valuable.

